# Supplementary material for: Mechanisms of Caspases 3/7/8/9 in the Degeneration of External Gills of Chinese Giant Salamanders (Andrias davidianus)
Source: Genes (Basel). 2022 Jul 29;13(8):1360. doi: 10.3390/genes13081360 (PMC9407298; doi:10.3390/genes13081360)
Supplement: Supplementary file 1 [file genes-13-01360-s001.zip › genes-1838327-supplementary.pdf]

Supporting information

Table S1. Information on sequences used in multiple sequence alignment.

Species	Accession number
**Caspase3**	
Mus musculus	AAC53196.1
Homo sapiens	CAC88866.1
Danio rerio	AAH78310.1
Xenopus laevis	NP_001081225.1
Oryzias latipes	NP_001098140.1
Xenopus tropicalis	NP_001120900.1
Ictalurus punctatus	NP_001188010.1
Alligator sinensis	XP_025063386.1
Microcaecilia unicolor	XP_030047368.1
**Caspase7**	
Xenopus tropicalis	NP_001016299.1
Danio rerio	NP_001018443.1
Chrysemys picta bellii	XP_005293940.1
Homo sapiens	XP_016872253.1
Nanorana parkeri	XP_018419982.1
Rhinatrema bivittatum	XP_029466303.1
Microcaecilia unicolor	XP_030058570.1
Thamnophis elegans	XP_032081709.1
Mus musculus	XP_006526679.3
**Caspase8**	
Mus musculus	NP_033942.1
Danio rerio	NP_571585.2
Oryzias latipes	NP_001098258.1
Oncorhynchus mykiss	NP_001268251.1
Homo sapiens	XP_005246949.1
Chrysemys picta bellii	XP_005306306.1
Rhinatrema bivittatum	XP_029462103.1
Xenopus tropicalis	XP_017953067.2
Chelonia mydas	XP_037768405.1
**Caspase9**	
Homo sapiens	P55211.3
Mus musculus	CAJ18606.1
Oncorhynchus mykiss	NP_001118119
Danio rerio	NP_001007405.2
Chrysemys picta bellii	XP_005293057.1
Nanorana parkeri	XP_018424267.1
Rhinatrema bivittatum	XP_029434788.1
Microcaecilia unicolor	XP_030041780.1
Chelonia mydas	XP_037737237.1

Table S2. Information on sequences used in phylogeny.

Species	Accession number
**Caspase3**	
Anas platyrhynchos	XM_027456288.1
Capra hircus	NM_001286089.1
Chelonia mydas	XM_007054464.2
Chrysemys picta bellii	XM_005281973.3
Cynops orientalis	JQ320088.1
Dromaius novaehollandiae	XM_026105278.1
Gallus gallus	NM_204725.1
Homo sapiens	NM_004346.4
Ictalurus_punctatus	XM_017473312.1
Microcaecilia unicolor	XM_030191508.1
Mus musculus	XM_017312543.3
Oncorhynchus mykiss	AB477342.1
Ornithorhynchus anatinus	XM_029076645.1
Oryzias latipes	NM_001104670.1
Parus major	XM_015624463.3
Protopterus annectens	XM_044089855.1
Protopterus_annectens	XM_044089854.1
Rana catesbeiana	BT081679.1
Takifugu rubripes	AY102865.1
Thamnophis elegans	XM_032224719.1
Xenopus laevis	NM_001097686.1
Xenopus laevis	XM_018234069.2
Xenopus laevis	XM_018251735.2
**Caspase7**	
Anas platyrhynchos	XM_027459864.2
Bufo gargarizans	XM_044296926.1
Bufo gargarizans	XM_044296925.1
Chrysemys picta bellii	XM_005293883.3
Cynoglossus semilaevis	XM_025057176.1
Cynops orientalis	JQ320087.1
Danio rerio	NM_001020607.1
Gallus gallus	XM_421764.5
Homo sapiens	XM_017016764.1
Microcaecilia unicolor	XM_030202710.1
Molothrus ater	XM_036385684.1
Mus musculus	NM_007611.2
Nanorana parkeri	XM_018564480.1
Orcinus orca	XM_004265822.2
Oryctolagus cuniculus	XM_017348571.1
Pogona vitticeps	XM_020779907.1
Protopterus annectens	XM_044055765.1

Pseudonaja textilis	XM_026704804.1
Rhinatrema bivittatum	XM_029610443.1
Xenopus laevis	NM_001097803.1
Xenopus laevis	NM_001087939.1
Xenopus laevis	XM_041568399.1
Xenopus tropicalis	NM_001016299.2
Xenopus tropicalis	NM_001016299.2(2)
Xenopus tropicalis	BC170775.1
Xenopus tropicalis	BC170801.1
Xenopus tropicalis	XM_031905081.1
Xenopus tropicalis	XM_031905082.1
**Caspase8**	
Catharus ustulatus	XM_033065450.1
Chelonia mydas	XM_037912477.1
Chrysemys picta bellii	XM_005306249.3
Danio rerio	NM_131510.2
Gallus gallus	NM_204592.3
Homo sapiens	XM_005246892.2
Lynx canadensis	XM_030325757.2
Mus musculus	NM_009812.2
Oncorhynchus mykiss	NM_001281322.1
Oryzias latipes	NM_001104788.1
Oxyura jamaicensis	XM_035332072.1
Rhinatrema bivittatum	XM_029606243.1
Scophthalmus maximus	XM_035635876.1
Tursiops truncatus	XM_019939136.2
Xenopus laevis	NM_001085565.1
Xenopus tropicalis	XM_018097578.2
**Caspase9**	
Anas platyrhynchos	XM_038166520.1
Chelonia mydas	XM_037881309.1
Chrysemys picta bellii	XM_005293000.3
Danio rerio	NM_001007404.2
Gallus gallus	XM_424580.6
Homo sapiens	NM_032996.3
Microcaecilia unicolor	XM_030185920.1
Mus musculus	CT010400.1
Oncorhynchus mykiss	NM_001124647.1
Oncorhynchus nerka	XM_029622430.1
Ornithorhynchus anatinus	XM_029066662.1
Podarcis muralis	XM_028740558.1
Scophthalmus maximus	XM_035644988.1
Serinus canaria	XM_018924683.2
Vombatus ursinus	XM_027859817.1

Xenopus laevis	NM_001085566.1
Xenopus tropicalis	NM_001123463.1
Xenopus tropicalis	XM_031904803.1

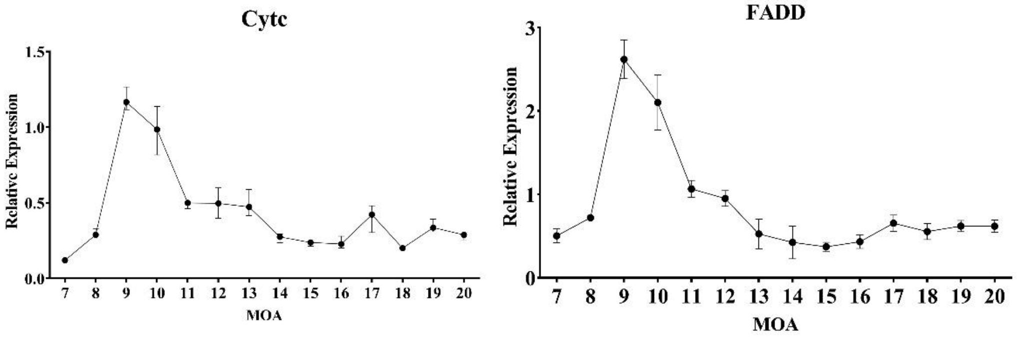

Figure S1. The expression trend of FADD and Cytc genes in the external gill of Chinese giant salamander during metamorphosis
